# Supplementary material for: A 1-Hydroxy-2,4-Diformylnaphthalene-Based Fluorescent Probe and Its Detection of Sulfites/Bisulfite
Source: Molecules. 2021 May 21;26(11):3064. doi: 10.3390/molecules26113064 (PMC8196617; doi:10.3390/molecules26113064)
Supplement: Supplementary file 1 [file molecules-26-03064-s001.zip › molecules-1195862-supplementary.pdf]

Supporting Information

A 1-Hydroxy-2,4-Diformylnaphthalene-Based Fluorescent Probe and Its Detection of Sulfites/Bisulfite

Qing Shi [1], Ling-Yi Shen [2], Hong Xu [1,2], Zhi-Yong Wang [2], Xian-Jiong Yang [2], Ya-Li Huang [2,*], Carl Redshaw [3] and Qi-Long Zhang [1,2,*]

1 School of Public Health, The Key Laboratory of Environmental Pollution Monitoring and Disease Control, Ministry of Education, Guizhou Medical University, Guiyang 550004, China; shiqing5900@126.com (Q.S.); xuhong@gmc.edu.cn (H.X.)

2 School of Basic Medical Science, Guizhou Medical University, Guiyang 550004, China; shenly@stumail.nwu.edu.cn (L.-Y.S.); zql810921@163.com (Z.-Y.W.); yangxianjiong@126.com (X.-J.Y.)

3 Department of Chemistry, University of Hull, Cottingham Road, Hull HU6 7RX, UK; c.redshaw@hull.ac.uk

* Correspondence: ylh6401@gmc.edu.cn (Y.-L.H.);gzuqlzhang@126.com (Q.-L.Z.); Fax: +86-0851-88174017 (Q.-L.Z.)

Contents:

HRMS of probe L **S1**

^{1}H NMR spectrum of probe L **S2**

^{13}C NMR spectrum of probe L **S3**

The effect of reaction time on detection **S4**

Photophysical properties of L **S5-S6**

X-ray crystallographic analysis of L **Table S1**

The influence of pH on stability of detection **Table S2-S3**

Comparison data with reported SO_3^{2-}/HSO_3^- sensors **Table S4**

High Resolution Mass Spectroscopy

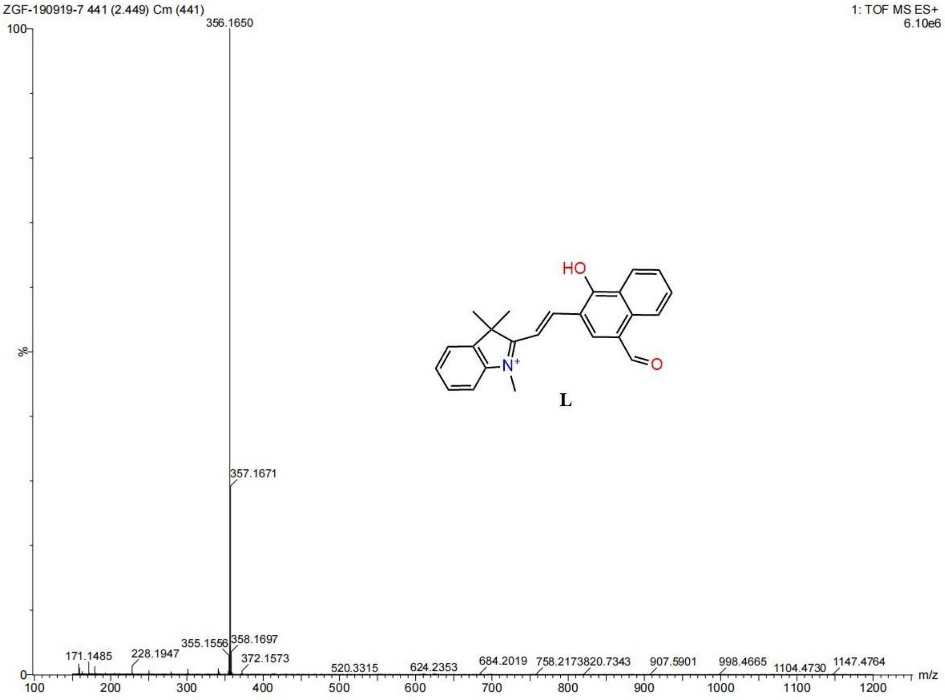

Figure S1. HRMS spectrum of probe **L**

NMR spectrum

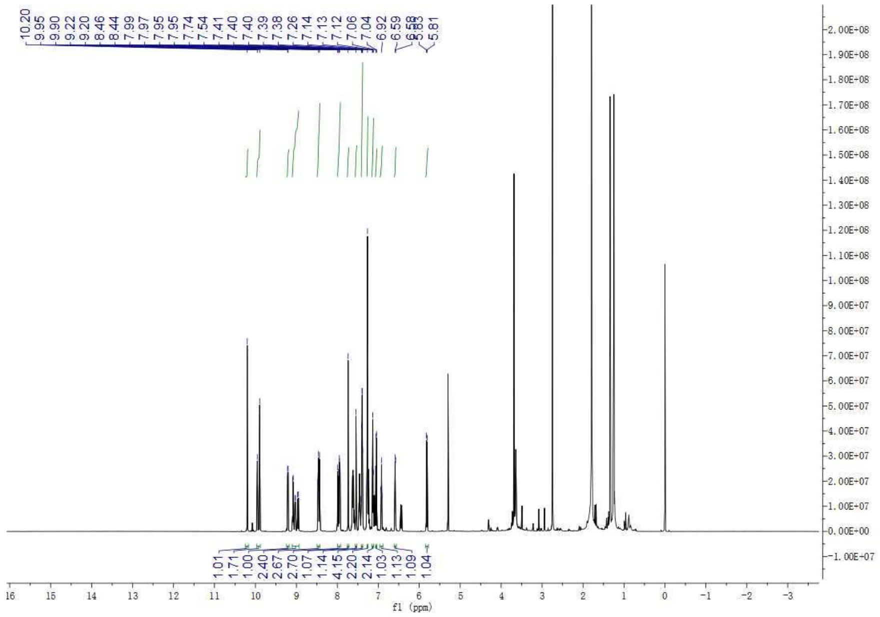

Figure S2. ^{1}H NMR of probe **L**

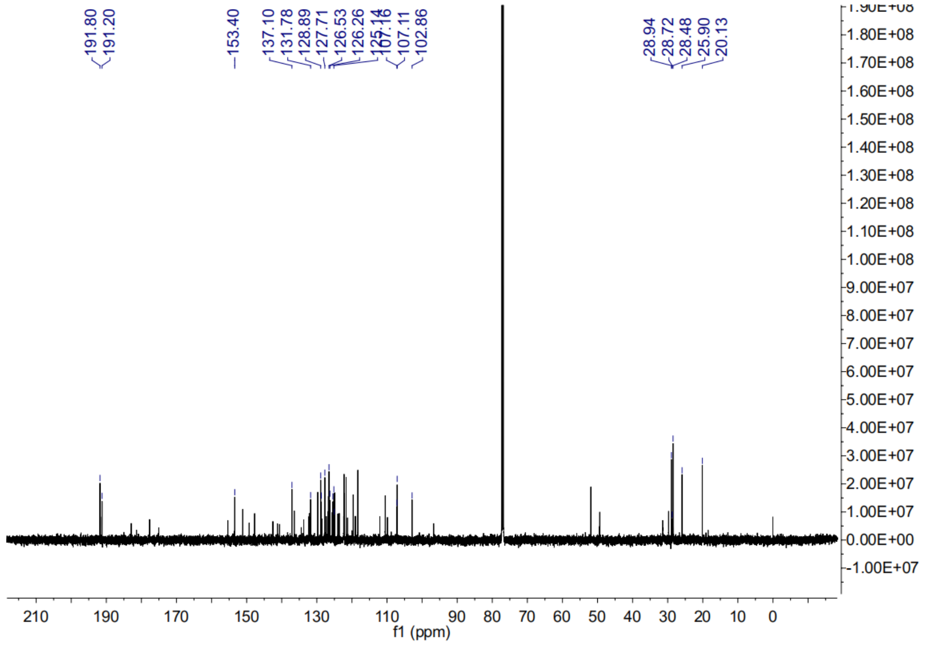

Figure S3. ^{13}C NMR of probe **L**

The effect of reaction time on detection

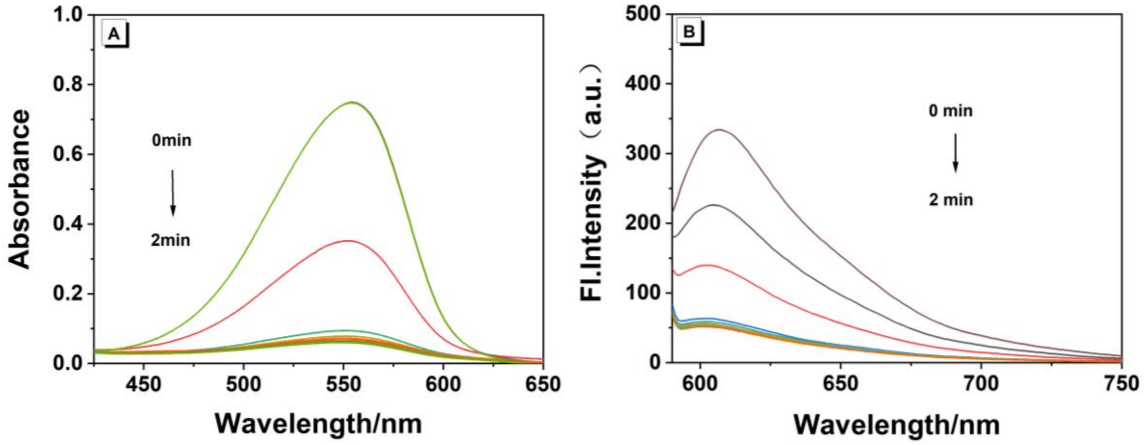

Figure S4. Uv-vis absorption spectra (A) and fluorescence spectra (B) of probe **L** (15 μM) in EtOH/water (V_{EtOH}/V_{water}=2/3, pH=7.40) after adding SO_3^{2-}/HSO_3^- (750 μM) over time ($\lambda_{ex}/\lambda_{em}$ = 576/605 nm, slit: 5/5 nm, voltage: 600 v)

Photophysical Properties

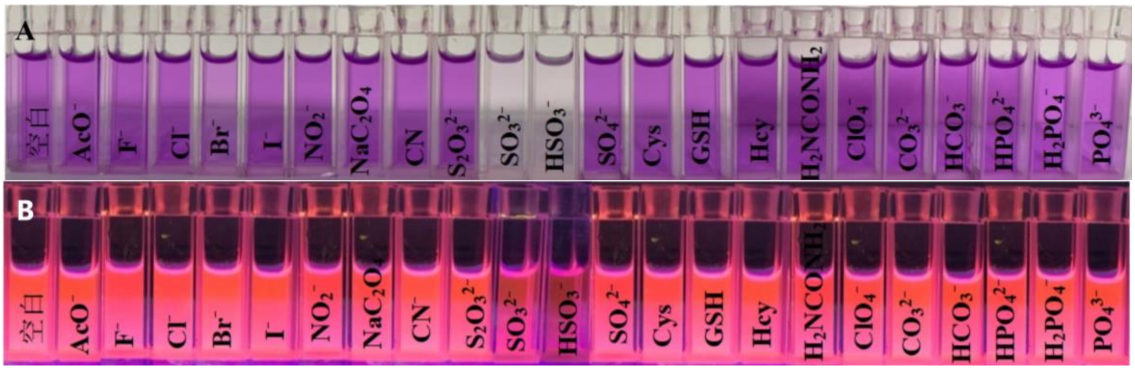

Figure S5. Photographs of probe **L**-anion complex in EtOH/water (V_{EtOH}/V_{water}=2/3, pH=7.40) solution under (A) natural light and (B) 365 nm UV lamp.

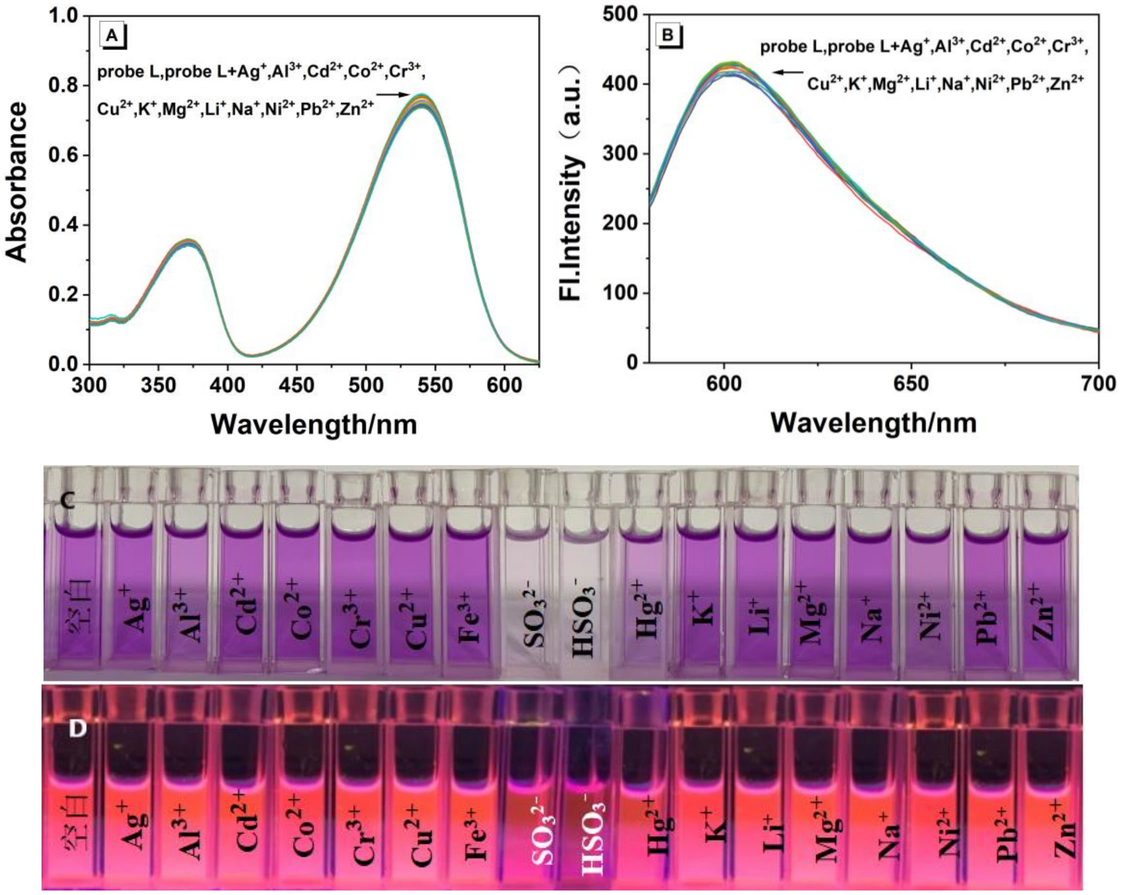

Figure S6. (A) UV-vis and (B) Fluorescence spectra of the fluorescence probe **L** interacting with different cations ($\lambda_{ex}/\lambda_{em}$ = 576/605 nm, slit: 5/5 nm, voltage: 600 v). Photographs of probe **L**-cation complex in EtOH/water (V_{EtOH}/V_{water}=2/3, pH=7.40) solution under (A) natural light and (B) 365 nm UV lamp.

X-ray crystallography Analysis

Table S1. Summary of crystal data of probe **L**

Parameter	Probe
Empirical formula	$C_{25} H_{21}NO_3$
Formula weight [g mol^{-1}]	383.43
Crystal system	monoclinic
Space group	$P2_1/c$
a [Å]	15.018(3)
b [Å]	7.5680(12)
c [Å]	18.180(3)
β [°]	99.278(4)
Volume [Å^3]	2039.2(6)
Z	4
Density, calcd [gm^{-3}]	1.249
Temperature [K]	300(2)
F(000)	808
Unique reflns	1543
Obsdreflns	*3573*
Parameters	267
R_{int}	0.0900
$R[I>2\sigma(I)]^a$	0.0811
W[all data]R^b	0.1857
GOF on F^2	0.904

[a] Conventional R on F_{hkl}: $\Sigma||F_o|-|F_c||/\sigma|F_o|$. [b] Weighted R on $|F_{hkl}|^2$: $\Sigma[w(F_o^2-F_c^2)^2]/\Sigma[w(F_o^2)^2]^{1/2}$.

The influence of pH on stability of detection

Table S2. The absorbance of probe L and L-SO_3^{2-}/HSO_3^- complex *versus* different pH value within 1440 mins (550 nm).

	pH value	Time			
		0 min.	360 min.	720 min.	1440 min.
Probe L	3	0.8756	0.8694	0.8573	0.8452
	4	0.8839	0.8742	0.8618	0.8508
	5	0.8846	0.8740	0.8653	0.8545
	6	0.8721	0.8609	0.8542	0.8427
	7	0.8783	0.8694	0.8537	0.8489
	8	0.8740	0.8656	0.8571	0.8436
	9	0.8854	0.8709	0.8634	0.8507
	10	0.8635	0.8578	0.8467	0.8402
	11	0.8678	0.8543	0.8481	0.8420
L-SO_3^{2-}/HSO_3^- complex	3	0.0836	0.0823	0.0816	0.0809
	4	0.1218	0.1205	0.1196	0.1192
	5	0.1945	0.1939	0.1924	0.1911
	6	0.1877	0.1873	0.1860	0.1855
	7	0.1928	0.1922	0.1903	0.1904
	8	0.1723	0.1717	0.1709	0.1701
	9	0.1726	0.1721	0.1716	0.1705
	10	0.1890	0.1890	0.1890	0.1890
	11	0.1424	0.1420	0.1415	0.1408

Table S3. The fluorescence intensity (a.u.) of probe L and L-SO_3^{2-}/HSO_3^- complex *versus* different pH value within 1440 mins (605 nm, slit: 5/5 nm, voltage: 800 v).

	**pH value**	**Time**			
		**0 min.**	**360 min.**	**720 min.**	**1440 min.**
	3	**769.1422**	**769.1347**	**769.1314**	**769.1308**
	4	**747.2058**	**747.2034**	**747.2018**	**747.1980**
	5	**745.0009**	**744.9649**	**744.9372**	**744.9245**
	6	**735.3779**	**735.3698**	**735.3650**	**735.3595**
**Probe L**	7	**736.7302**	**736.7276**	**736.7235**	**736.7179**
	8	**727.0831**	**727.0648**	**727.0473**	**727.0134**
	9	**710.4871**	**710.4547**	**710.4481**	**710.4423**
	10	**702.2395**	**702.2350**	**702.2324**	**702.2316**
	11	**701.4871**	**701.4769**	**701.4743**	**701.4609**
	3	**54.3564**	**54.3475**	**54.3416**	**54.3409**
	4	**81.4291**	**81.4235**	**81.4217**	**81.4196**
	5	**120.8952**	**120.8934**	**120.8926**	**120.8895**
	6	**116.8050**	**116.8025**	**116.7959**	**116.7943**
**L-SO_3^{2-}/HSO_3^- complex**	7	**122.9310**	**122.9277**	**122.9204**	**122.9126**
	8	**112.0084**	**112.0036**	**111.9805**	**111.9695**
	9	**112.8395**	**112.8347**	**112.8328**	**112.8108**
	10	**120.5730**	**120.5635**	**120.5607**	**120.559**
	11	**130.8527**	**130.8504**	**130.8497**	**130.8449**

Table S4. Comparison data with reported SO_3^{2-}/HSO_3^- sensors

Structure	Response time	Fluorescence	solvent	Detection limit
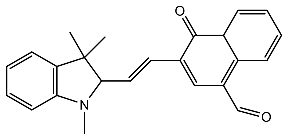 **Our work**	2 min.	Turn-off	EtOH/ H_2O (2:3)	0.24 μM/9.93 nM
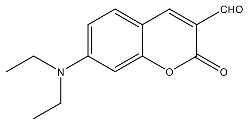 **C1 [1]**	2 min.	Turn-on	THF/H_2O (1:99)	3.00 μM
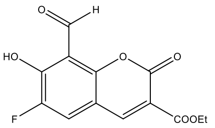 **3b [2]**	5 min.	Turn-on	NaH_2PO_4 Citric (pH=5)	0.37 μM
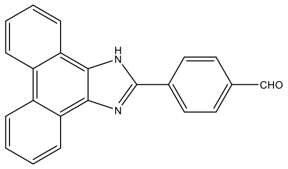 **a1 [3]**	10 min.	Turn-on	THF/H_2O (1:99)	2.00 μM
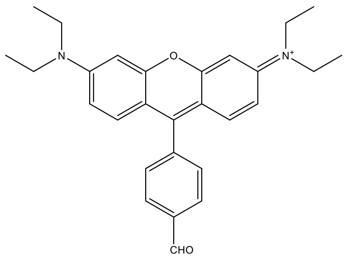 **RosCHO [4]**	10 min.	Turn-on	Tris-HCl (pH=5)	0.07 μM

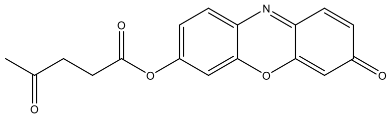 **Probe 1 [5]**	20 min.	Turn-on	CH_3CN/H_2O (2:98)	49.00 μM
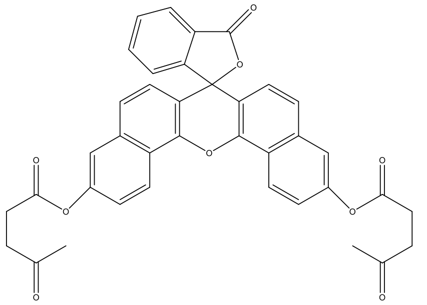 **Probe 1 [6]**	20 min.	Turn-on	DMSO/ H_2O (1:1)	1.74 μM
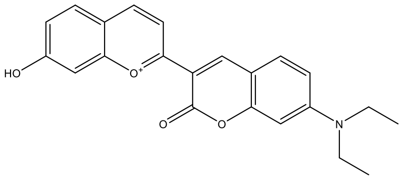 **Probe 1 [7]**	5 min.	Turn-on	$EtOH/H_2O$ (3:7)	34.00 nM
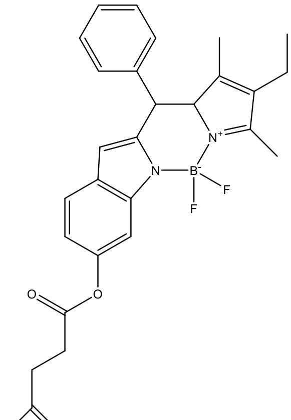 **BODIPY-Le [8]**	20 min.	Turn-off	DMSO/ H_2O (1:1)	58.00 μM

References

1. Cheng, X. H.; Jia, H. Z.; Feng, J.; Qin, J. G.; Li Z., "Reactive"probeforhydrogensulfite: "turn-on" fluorescent sensing and bioimaging application. *Journal of Materials Chemistry B* **2013**, *1*(33), 4110-4114.

2. Chen, K. Y.; Guo, Y.; Lu, Z. H.; Yang, B. Q.; Shi, Z., Novel Coumarin-based Fluorescent Probe for Selective Detection of Bisulfite Anionin Water. *Chinese Journal of Chemistry* **20l0**, *28*(1), 55-60
3. Zhang, D.; Liu, W. Y.; Chen, K. K.; Cheng, J. Y.; Zhao, Y. F; Ye, Y., A novel rosamine-based fluorescent probe for bisulfite in aqueoussolution. *The Royal Society of Chemistry* **2016**, *6*(106), 103905-103909.
4. Choi, M. G.; Hwang, J.; Eor, S.; Chang, S-K., Chromogenic and fluorogenic signaling of sulfite by selective deprotection of resorufin levulinate. *Organic Letters* **2010**, *l2*(24), 5624-5627.
5. Zhang, H. Y.; Xue, S. H.; Feng, G. Q., A colorimetric and near-infrared fluorescent turn-on probe for rapid detection of sulfite. *Sensors and Actuators, B-Chemical* **2016**, *231*, 752-758.
6. Chern, Y. H.; Wang, X.; Yang, X. F.; Zhong, Y.; Li, Z.; Li, H., Development of a ratiometric fluorescent probe for sulfite based on a coumarin-benzopyrylium platform. *Sensors and Actuators B: Chemical* **20l5**, *206*(206), 268-275.
7. Gu, X. F.; Liu, C. H.; Zhu, Y. C.; Zhun, Y. Z., A Boron-dipyrromethene-Based Fluorescent Probe for Colorimetric and Ratiometric Derection of Sulfite. *Journal of Agricultural and Food Chemistry* **2011,** *59*(221), l1935-11939.
